# Examining the Exercise and Self-Esteem Model Revised with Self-Compassion among Hong Kong Secondary School Students Using Structural Equation Modeling

**DOI:** 10.3390/ijerph18073661

**Published:** 2021-04-01

**Authors:** Ming-Yu Claudia Wong, Pak-Kwong Chung, Ka-Man Leung

**Affiliations:** 1 Department of Sport, Physical Education and Health, Hong Kong Baptist University, Kowloon, Hong Kong; 18481795@life.hkbu.edu.hk; 2 Department of Health and Physical Education, Education University of Hong Kong, New Territories, Hong Kong; leungkaman@eduhk.hk

**Keywords:** physical activity, self-compassion, adolescence, exercise and self-esteem model, mental health

## Abstract

The association between physical activity in achieving mental health benefits and subjective well-being is consistently identified by empirical research. The causation of a positive self-concept created by physical exercise is empirically supported by Sonstroem and Morgan’s (1988) exercise and self-esteem model (EXSEM). However, various drawbacks of maintaining high self-esteem have been identified; thus, the concept of self-compassion was conjectured to be a form of “true self-esteem.” Hence, the current study aimed to investigate the relationship between physical activity and self-compassion by examining the exercise and self-esteem model revised with self-compassion (EXSEM-SC). This study recruited secondary school students from Hong Kong using convenience sampling. The structural equation modeling (SEM) approach, including path analysis and multiple indicators, multiple causes (MIMIC) modeling, were used to reveal the results of the study. The results (*n* = 1097) indicated that the relationship between physical activity and self-compassion could be demonstrated by the EXSEM-SC, with a satisfactory goodness-of-fit index in the SEMs. The SEM also demonstrated the direct paths from physical activity to self-compassion and mental well-being, indicating the significant effect of physical activity on self-compassion.

## 1. Introduction

Mental health is defined as “a state of well-being in which an individual realizes his or her own abilities, copes with the normal stresses of life, works productively, and is able to make a contribution to his or her community [1]” while comprising collective conditions. The mental health problems of children and adolescents in Hong Kong are seen as serious and should be considered as the public’s main focus. In Hong Kong, there was a significant drop in the overall Children’s Happiness Index for two consecutive years, from 6.91 in 2012 to 6.74 in 2014 to 6.49 in 2015. The Hong Kong University (HKU) Centre for Suicide Research and Prevention (2018) [2] highlighted that the suicide rate of full-time students aged 15–24 years increased by 76.1%. Despite the decrease in the proportion of suicide students with a psychiatric history, the investigation showed that most of the influencing problems were related to family, romantic relationships, study, and finance, which might also lead to mental health issues or emotional problems.

### 1.1. Physical Activity and Mental Health

The World Health Organization (WHO) has once documented that the lack of work-life balance leads to mental illness, mainly as high levels of stress, anxiety, and depression, while engaging in physical activities is considered a way to prevent and release negative psychological symptoms [3,4,5]. Studies have shown that students with a higher level of physical activity were associated with a lower risk of academic burnout; however, this was also related to individuals’ mental toughness [6]. Along with the specificity of the association between physical activity and students’ academic burnout, various mental health outcomes have been examined and investigated, such as the influences on mood, self-esteem, cognition, depression, and anxiety [7]. Various systematic reviews and meta-analyses have also demonstrated the effectiveness and significance of physical activity in improving adolescents’ mental health and reducing mental illness, such as health-related quality of life, self-esteem, depression, anxiety, and other psychosocial outcomes [8,9,10].

### 1.2. Physical Activity and Positive Self-Concept

The association between physical activity in achieving mental health benefits and subjective well-being is consistently identified by empirical research. The causation of positive self-concept brought by physical exercise is also empirically supported [11,12,13]. This bottom-up causal relationship is consolidated and structured by Sonstroem and Morgan’s (1988) [14] exercise and self-esteem model (EXSEM) based on Shavelson, Hubner, and Stanton’s (1976) [15] self-concept structure. The EXSEM is a popular bottom-up model in examining exercise intervention and in investigating the dose-effects of physical activity on the self from changing physical self-perception to enhancing global self-esteem [16,17,18]. Nonetheless, a recent meta-analysis showed insignificant causality between physical activity and self-esteem [8,19].

### 1.3. Self-Compassion and Drawbacks of Self-Esteem

Self-esteem is considered a symbolic concept that illustrates the psychological stance of oneself. Studies have reported the association of high self-esteem with psychological benefits and high self-esteem. However, Neff (2003, 2011) [20,21] identified various drawbacks of maintaining high self-esteem. It is concerning that high self-esteem is associated with self-enhancement bias [22], whereby people overrate themselves. Other than having a biased self-evaluation, individuals with extensive high self-esteem would have a strong sense of social comparison leading to narcissism, self-centeredness, and other negative mentalities [23,24]. Therefore, self-compassion is seen as providing equilibrium mental benefits and preventing the risk of extreme positive psychological status.

Studies have demonstrated that self-compassionate individuals are more likely to engage in positive thinking, have greater self-awareness, and appraise themselves more accurately [25,26,27]. Moreover, studies on self-compassion intervention have demonstrated effective gains in mental well-being, including mindfulness, life satisfaction, connectedness, optimism, self-efficacy, rumination (inversely), and worry (inversely) [28], thus leading to reduced levels of depression [29]. Self-compassion has been shown to mediate between college students’ negative life events and suicide risks [30]; hence, self-compassion provided them with emotional benefits and a higher level of engagement in wellness behaviors [31,32].

Deci and Ryan conjectured that self-compassion could be a form of “true self-esteem” [26,27,33] that created higher stability of self-worth when compared with global self-esteem. A longitudinal study revealed that self-esteem was anterior to the development of self-compassion, but not vice versa [34], hence, self-compassion was evoked to prevent high self-esteem, which also avoids leading to egoistic illusion and self-regulation failure [35]. On the one hand, self-compassion was proved to be associated with physical-related self-esteem, like appearance self-worth and physical self-perception [36,37]. On the other hand, numerous researchers have indicated that self-compassion can be significantly distinguished from self-esteem and even results in more comprehensive outcomes in predicting both negative and positive mental well-being, as well as ego-focused activity. Research indicated that the partial correlation between self-compassion and anxiety was still significant after the controlling of self-esteem and have shown additional variance after adding self-compassion in the hierarchal regression analysis of positive mental well-being [27,38,39].

Together with a recent systematic review and meta-analysis, self-compassion was significantly associated with physical activity and physical activity intention [40]. Self-compassion should be proposed as a universal and extensive construct distinct from global self-esteem [27,39]. Hence, the current study aimed to investigate the relationship between physical activity and self-compassion by modifying the EXSEM with self-compassion.

### 1.4. Hypothesized Exercise and Self-Esteem Model (EXSEM) Revised with Self-Compassion

**Figure 1:**
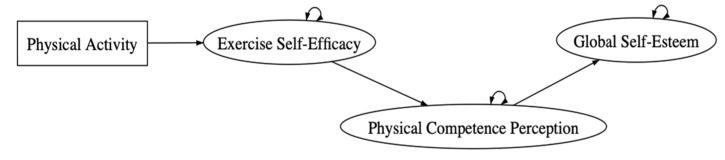
The original exercise and self-esteem model (EXSEM).

The proposed EXSEM revised with self-compassion was structured according to the original EXSEM (Figure 1). In this study, a pathway leading to self-compassion was proposed. Hence, the exogenous variable (physical activity) and the expected mediating variable (exercise self-efficacy) are maintained.

**Figure 2:**
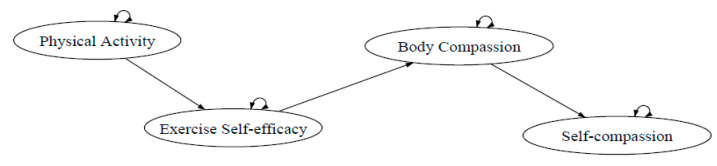
The Exercise and self-esteem model revised with self-compassion (EXSEM-SC).

The proposed model replaced physical competence perception and global self-esteem with body compassion and self-compassion, respectively, as shown in Figure 2. It is noteworthy that the perception of the physical self (physical competence and acceptance), a mediating variable, was originally accessed by the Physical Self-Perception Profile. In the revised model, body compassion [41] was used to demonstrate the physical self and act as the hypothesized mediating variable toward self-compassion. Body compassion was developed by including physical self and the concept of self-compassion (Altman et al., 2017); thus, it was expected to be a more distinctive method to demonstrate the level of physical self toward self-compassion. In addition, it showed a strong correlation with the Body Image-Acceptance and Action Questionnaire [41,42]. Hence, body compassion can be expected to be in line with Sonstroem and Morgan’s (1988) [14] concept of physical self, which included the measurement of self-acceptance with the body, its function, how it looks, and ability.

**Figure 3:**
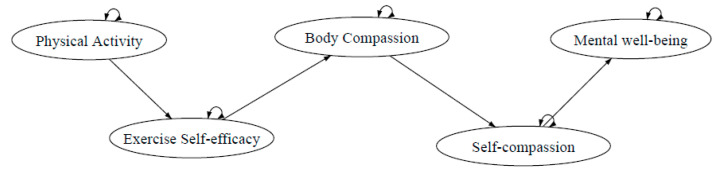
Extension of exercise and self-esteem model revised with self-compassion (EXSEM-SC) including mental well-being.

In addition, to further indicate the effect of physical activity and self-compassion on individuals’ mental well-being, an extension of the hypothesized model (Figure 3) with the mental health variable was developed for investigation.

## 2. Materials and Methods

### 2.1. Participants and Sampling

This study targeted secondary school students in Hong Kong. In response to the sample size rule of thumb of structural equation modeling, the sample size is suggested to be 10 times that of the variable indicators [43]. Therefore, according to the total number of variable indicators in the current study and a 70% expected response rate, a sample size of more than 1000 is expected.

In the current study, because of the unfavorable response from the stratified proportional randomly selected schools, convenience sampling was used to recruit participants. Finally, three schools from New Territories (*n* = 579), and one school each from Kowloon (*n* = 231) and Hong Kong Island (*n* = 285) were recruited. Moreover, with regard to age as a covariant within the mechanism, students from both junior (Form 1–3) and senior forms (Form 4–6) were recruited from each school. As several studies have indicated a significant difference in self-compassion levels in younger and older adolescents [44,45], the inclusion of both early and late adolescents can provide a possible confounder and achieve a better generalization of results.

### 2.2. Measures

#### 2.2.1. Self-Compassion

The Chinese version of the Self-compassion Scale [25,46] was used to measure the level of self-compassion. The scale consists of 26 items, of which the total score can determine the level of self-compassion of individuals. The 26 items were derived from the six self-compassion components: self-kindness, common humanity, mindfulness, self-judgment, isolation, and over-identification. Sample items include: “I’m disapproving and judgmental about my own flaws and inadequacies” (reverse coding) and “When something upsets me, I try to keep my emotions balanced.” The items were rated on a 5-point scale (1 = almost never, 5 = almost always). The scale also showed a Cronbach’s alpha of over 0.80 in the Chinese students’ population and with the test-retest reliability at 0.89 [46], which verified that the psychometric quality of the Chinese version of Self-compassion Scale was adequate.

#### 2.2.2. Body Compassion

The Chinese-translated body compassion scale was used to measure the level of body compassion. The body compassion scale is a construct that represents a combination of physical-related attitude and well-being at a mindfulness and self-acceptance approach, in other words, enlightening both physical perception and self-compassion. The scale items were created according to the three subscales of the Self-compassion Scale: mindfulness versus over-identification, self-kindness versus self-judgment, and common humanity versus isolation [25]. Each subscale item also corresponded with the three major components of the physical self (appearance, competence, and health) [14,47]. The body compassion scale is a 23-item scale with three subscales: defusion, common humanity, and acceptance. The items were rated on a 5-point scale (1 = almost never, 5 = almost always) [25,41]. The level of body compassion is calculated by summing up the score of common human, acceptance, and the reverse-scored defusion subscale, with scores ranging from 26 to 114. The Chinese-translated body compassion scale [48] showed adequate psychometric properties, with satisfactory internal consistency and test-retest reliability. It showed adequate goodness-of-fit results in construct validity, with X^²^ (465.64)/227 = 2.05, *p* < 0.001, comparative fit index (CFI) = 0.916, Tucker–Lewis index (TLI) = 0.906, standardized root mean square residual (SRMR) = 0.071, root mean square error of approximation (RMSEA) = 0.069 (90% confidence interval (CI) = 0.06 to 0.078).

#### 2.2.3. Exercise Self-Efficacy

The Exercise Self-efficacy Scale [49,50] was adopted. The Chinese version is a 17-item, 7-point Likert-type scale modified from that of Bandura (2006). The statements required the respondents to rate how certain (or confident) they were to engage in their exercise routine regularly under a range of conditions, such as when they are tired, when in bad weather, or when there are other special conditions. The original scale is an 18-item scale and uses a 10-unit interval, ranging from 0 (cannot do) to 100 (highly certain they can do). However, within the Chinese population, the 18-item scale was evaluated and modified, resulting in 17 items. The Chinese version has adopted the 7-point Likert scale that ranges from 0 to100, with 1 representing “cannot do at all” and 7, “highly certain can do” [51,52]. In the same studies, the evaluation of the Chinese version of the scale revealed a Cronbach’s alpha value of 0.944, which indicated satisfactory reliability.

#### 2.2.4. Physical Activity Subjective Measure

The Chinese version of the Physical Activity Questionnaire for Adolescents (PAQ-A) was utilized to obtain self-reported physical activity levels. The PAQ-A is a subjective measure of adolescents’ physical activity. It consisted of nine questions on a 5-point Likert scale, eight of which were used to calculate moderate to vigorous physical activity. The PAQ-A is self-administered and asks respondents to recall their physical activity in the past week (seven days) to measure the average moderate to vigorous physical activity levels of high school students. The PAQ-A questionnaire has shown relatively moderate-to-strong correlations with other physical activity measurement scales, including the International Physical Activity Questionnaire (IPAQ) [53]. The Chinese version of PAQ-A was also adopted and evaluated among adolescents aged 13–14 years and achieved a result of Cronbach’s alpha ranged from 0.82 to 0.85, with a 0.81 test-retest reliability after omitting question number 3 (lunchtime activity). In the same study, the objective measure of physical activity has also been applied as a validity checking tool and has revealed a moderate correlating coefficient with the total physical activity and moderate and vigorous activity levels [54].

#### 2.2.5. Mental Well-Being

The Chinese version of the Warwick–Edinburgh Mental Well-being Scale Short Form (SWEMWBS) [55] was adopted to measure individuals’ positive mental well-being life in various aspects. This scale includes affective-emotional, cognitive-evaluative, and psychological function items in a short and simple format [56]. The short version of the WEMWBS is a 7-item scale; sample items include, “I have been feeling optimistic about the future” and “I have been feeling useful.” The items are rated on a 5-point Likert scale (1 = none of the time, 2 = rarely, 3 = some of the time, 4 = often, and 5 = all the time). The psychometric properties validation results of the Chinese version demonstrated a satisfactory internal reliability of 0.93 and adequate test-retest reliability with a Cronbach’s alpha of 0.84. This also resulted in adequate concurrent validity [55]. In addition, the 7-item scale’s psychometric properties examination among Chinese students demonstrated a satisfactory internal consistency of 0.884 and a good model fit index, with CFI 0.986 and TLI = 0.979 [57].

### 2.3. Statistical Analysis

The SPSS 26 software was used to record the demographic information of the participants, including age and gender. Descriptive statistics on the level of physical activity, type of physical activity engagement, and level of self-compassion were synthesized to address the current phenomenon and for further analysis. The Pearson Correlation and the SPSS Process Macro by Andrew Hayes were used to indicate the basic association and the mediational relationships between the variables. Using the RStudio [58] the models were constructed and tested using a SEM approach, including path analysis in EXSEM-SC (Figure 2), EXSEM-SC with the six-factors of self-compassion and the extended EXSEM-SC including mental health (Figure 3); as well as multiple indicators, multiple causes (MIMIC) modeling, with the full-information maximum likelihood estimator (FIML). Besides, to further examine the differences between ages in the model, the invariant model testing was conducted as well. The goodness of fit of the models was determined according to the following criteria: (1) chi-square ranging from 2 to 5, (2) comparative fit index (CFI) and Non-Normed Fit Index (NNFI) rated as 0.90 or above as a model of good fit [59]; (3) standardized root mean square residual (SRMR) value of 0.08 or below as a model of good fit [59]; and (4) root mean square error of approximation (RMSEA) value as 0.08 or below, with a 90% confidence interval that holds within this value considered as a model of good fit [60].

## 3. Results

### 3.1. Demographic Information

A total of 1097 students participated in the survey. While 20 cases with more than 20% missing data were deleted, three univariate and multivariate outlier cases were identified and deleted. The remaining missing data were replaced with the mean values of the items. The variables in the data set were normally distributed with no items greater than 3.3, or less than −3.3. Finally, a total of 1074 cases were included in the data analysis, of which 65% and 35% were men and women, respectively; and the mean age was 14.13 (standard deviation (SD) = 1.44). According to the gender distribution statistics provided by the Education Bureau (2019) [61], the gender distribution of the current sample was comparable with the Hong Kong secondary school students’ gender distribution, with approximately 56% of men and 44% of women. It is also worth noting that the distribution among young and older teenagers was approximately even (Table 1).

In general, Table 2 shows that Hong Kong secondary school students’ exercise self-efficacy, body compassion, self-compassion, and mental well-being were moderate to high, whereas they indicated that their physical activity level was low to moderate. Among the self-reported physical activities, walking (*m* = 4.32, *SD* = 1.22), running/jogging (*m* = 2.07, *SD* = 1.12), and basketball (*m* = 1.49, *SD* = 0.93) were considered the top three physical activities that secondary school students participated in. Participants also reported other physical activities that were not stated in the questionnaire; they were mainly different kinds of ball games, fencing, and going to the gym. Table 3 shows favorable results regarding the relationship between variables, indicating significant correlations between most of the variables, with *p* < 0.001, except income and age. Compiling the variables of EXSEM-SC, the mediational analysis indicated the significant mediation role of exercise self-efficacy and body compassion in the relationship of physical activity and self-compassion, with the standardized indirect effect (0.66)(0.31)(0.27) = 0.06. In addition, the independent *t*-tests showed no significant differences between gender, social class, or age group in the level of self-compassion. However, they showed significant differences between gender and age group in the physical activity level, with t(1068) = 2.38, *p* = 0.02, and t(1055) = 4.07, *p* < 0.001. Owing to the significant differences shown, the moderating effects of age and gender were further tested. The moderating regression results demonstrated that gender had no moderating effect on the relationship between physical activity and self-compassion, while age had a significant moderating effect on the relationship between physical activity and self-compassion, with β = −0.093, t(209.94) = −3.039, *p* = 0.002. In other words, this indicates that despite the participants’ high level of physical activity, their level of self-compassion was reduced with an increase in age.

### 3.2. Confirmatory Factor Analysis of the Measurement Models

Physical Activity Questionnaire for Adolescents (PAQ-A) Chinese Version.

Table 4 presents the confirmatory factor analysis (CFA) results of the measurement models. The Cronbach’s alpha value of the PAQ-A Chinese version was 0.839, and the Omega value at 0.85. The factor analysis results demonstrated a Kaiser–Mayer–Olkin (KMO) value of 0.912. The initial measurement model showed a good fit index, with X^2^(110.12/20) = 5.5, CFI = 0.98, TLI = 0.978, SRMR = 0.028, RMSEA = 0.065 (0.054–0.077). To further improve the measurement model, the result of the CFA model, including the subsequent modification indices suggested covariance between “time spent on different types of physical activity per week” and “time spent on physical activity each day per week” and between the “number of times in the evening doing physical activity per week” and the “number of times doing physical activity during weekends,” showed an adequate goodness-of-fit measurement model, with X^2^ (38.85)/18 = 2.15, CFI = 0.995, TLI = 0.992, SRMR = 0.020, and RMSEA = 0.033 (90% CI = 0.019–0.047).

### 3.3. Exercise Self-Efficacy Scale-Chinese Version

The Cronbach’s alpha value and the Omega value of the Exercise Self-Efficacy Scale–Chinese version were both at 0.95. The factor analysis results demonstrated a KMO value of 0.997. The initial measurement model of exercise self-efficacy showed an inadequate goodness of fit in the chi-square value, with X^2^(1023.6/119) = 8.6, CFI = 0.92, TLI = 0.91, SRMR = 0.038, and RMSEA = 0.084(0.079–0.089). Hence, the result of the CFA model, including the subsequent modification indices suggested covariance between “feeling tired,” “feeling stress,” “feeling desperate,” “want to be lazy,” “having not enough time,” “without an exercise partner,” “without exercise equipment and facilities,” “without support from family or friends,” “have not been exercising for a long time.” and “when forcing yourself to do exercise,” showed an adequate goodness-of-fit measurement model, with X^2^(645.88)/113 = 5.7, CFI = 0.954, TLI = 0.945, SRMR = 0.031, and RMSEA = 0.066 (90% CI = 0.061–0.071).

### 3.4. Body Compassion Scale-Chinese Version

The Cronbach’s alpha value of the body compassion scale-Chinese version was 0.81, while the Omega value was 0.67. The factor analysis results demonstrated a KMO value of 0.93. The results of the CFA model showed a satisfactory goodness-of-fit measurement model, with X^2^(995.19)/227 = 3.36, CFI = 0.944, TLI = 0.938, SRMR = 0.042, and RMSEA = 0.056 (90% CI = 0.053–0.06). Additionally, a second-order CFA was conducted to confirm the theoretical construct of body compassion using the three subscales. The second-order CFA showed adequate goodness of fit, with X^2^(714.59)/204 = 3.5, CFI = 0.962, TLI = 0.957, SRMR = 0.037, and RMSEA = 0.048 (90% CI = 0.044–0.052).

### 3.5. Self-Compassion Scale-Chinese Version

The Cronbach’s alpha value and the Omega value of the Chinese version of the Self-compassion Scale were 0.83 and 0.71 respectively. The factor analysis results demonstrated a KMO value of 0.921. The initial six-factor solution measurement model showed an inadequate goodness-of-fit indices, with X^2^(1344.30/284) = 4.7, CFI = 0.898, TLI = 0.883, SRMR = 0.050, and RMSEA = 0.059 (0.056–0.062). Therefore, items 7 and 8 were deleted from the self-judgment and common humanity subscales, with low factor loadings of 0.260 and 0.266, respectively. The results of the CFA model showed a satisfactory goodness-of-fit measurement model, with X^2^(1020.67)/237 = 4.3, CFI = 0.918, TLI = 0.905, SRMR = 0.045, and RMSEA = 0.055 (90% CI = 0.052–0.059). Given that the second-order six-factor solution model of the self-compassion scale was reviewed to provide the best fit and representativeness in measuring self-compassion (Cunha et al., 2015; Neff et al., 2017), a second-order CFA was performed. The second-order CFA, with all subscales correlated, showed adequate goodness of fit, with X^2^(1020.66)/236 = 4.3, CFI = 0.918, TLI = 0.904, SRMR = 0.045, and RMSEA = 0.056 (90% CI = 0.052–059).

### 3.6. Warwick–Edinburgh Mental Well-Being Scale Short Form (SWEMWBS)-Chinese Version

The Cronbach’s alpha value and Omega value of the SWEMWBS-Chinese version were both 0.90. The factor analysis results demonstrated a KMO value of 0.910. However, the initial measurement model showed an inadequate goodness-of-fit index in the chi-square value and RMSEA, with X^2^(191.2/14) = 13.65, CFI = 0.96, TLI = 0.94, SRMR = 0.0034, and RMSEA = 0.109 (0.095–0.123). The results of the CFA model, with most of the items correlated, showed an adequate goodness-of-fit measurement model, with X² (42)/9 = 4.6, CFI = 0.920, TLI = 0.981, SRMR = 0.017, and RMSEA = 0.059 (90% CI = 0.042–0.77).

To conclude, the above results indicate the internal consistency, common variability between items, factorial validity, and construct validity of all the measurement models, which supports the construction of SEM.

### 3.7. Path Analysis

#### 3.7.1. Exercise and Self-Esteem Model Revised with Self-Compassion

**Figure 4:**
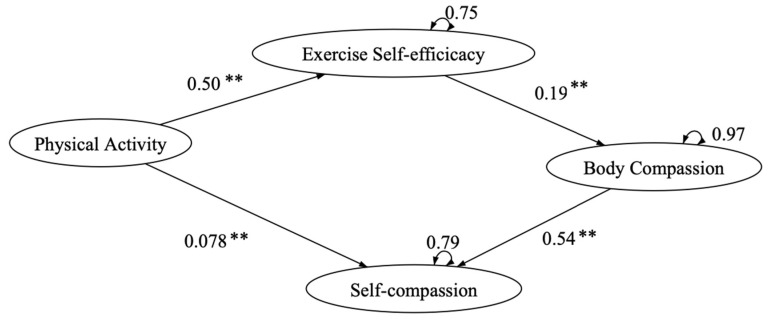
Exercise and self-esteem model revised with self-compassion. Note: ** *p* < 0.01.

To perform path analysis, the total scores of the measurement scales were computed for the observed variables. Based on the reviewed theoretical framework, the exercise and self-esteem model revised with self-compassion was proposed and used for statistical testing. The path analysis results demonstrated acceptable goodness of fit, with CFI = 0.915, TLI = 0.858, SRMR = 0.086, RMSEA = 0.121 (90% CI = 0.101–0.141), while all pathways were significant (*p* < 0.001). One direct pathway was identified from model modification. Apart from the original indirect pathways, physical activity to self-compassion was shown to be a significant direct pathway in the modified model. Hence, the final path model (Figure 4) has achieved an improved model fit, with X^2^(7.05)/2 = 3.5, CFI = 0.993, TLI = 0.979, SRMR = 0.018, and RMSEA = 0.049 (90% CI = −0.013–0.090). The pathways from physical activity to self-compassion are shown in Table 5.

#### 3.7.2. Exercise and Self-Esteem Model Revised with Six-Factor Model of Self-Compassion

Considering the multi-facet construct of self-compassion, the model with the effect of physical activity on the different facets of self-compassion was investigated. The path analysis results of the Exercise and Self-esteem Model Revised with Six-factor Model of self-compassion showed adequate goodness of fit index, with X^2^(76.11)/11 = 6.9, CFI = 0.993, TLI = 0.970, SRMR = 0.042, and RMSEA = 0.075 (90% CI = 0.06–0.09). The model paths showed significant indirect effects between physical activity and all the facets of self-compassion, ranging from β = 0.24–0.43. Also, it showed a significant direct effect between physical activity and the positive facets of self-compassion, including self-kindness (β = 0.07), common humanity (β = 0.07) and mindfulness (β = 0.12). While similar standardized solution value was shown in the other pathways. The results thus indicated the significant effect of physical activity towards positive self-compassion.

#### 3.7.3. Exercise and Self-Esteem Model Revised with Self-Compassion and Mental Health

**Figure 5:**
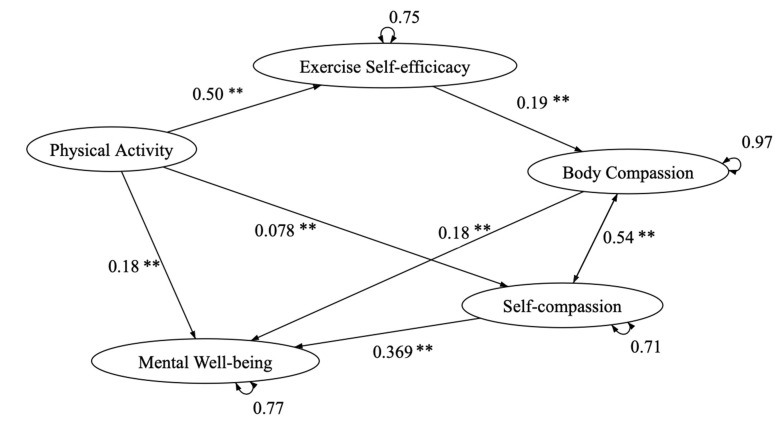
Exercise and self-esteem model revised with self-compassion and mental health. Note: ** *p* < 0.01.

To respond to the phenomenon of Hong Kong secondary school students’ mental health problems, mental health was added to the model for further examination. The path analysis results of the exercise and self-esteem model revised with self-compassion showed satisfactory model fit results for mental health, with X^2^(17.13)/3 = 5.67, CFI = 0.987, TLI = 0.955, SRMR = 0.028, and RMSEA = 0.068 (90% CI = 0.040–0.010). Furthermore, it showed two direct pathways from physical activity and body compassion to mental well-being (Table 6; Figure 5). Additionally, despite age seen as inferring a moderating effect between physical activity and self-compassion, the moderating path was seen as insignificant in the model.

#### 3.7.4. Measurement Invariant Model

To further examine the differences between age in the model, the invariant model testing was conducted using multiple-group SEM to examine whether the conceptual model was invariant across early and older adolescents. A baseline model without any constrained parameters was first established, and then two constrained models with constrained factor loadings (metric model) and constrained mean differences (scalar model) were established for equality across age groups. Table 7 demonstrates that the unconstrained model (baseline model) resulted in an adequate goodness-of-fit index for the data. Meanwhile, the metric and scalar models, with the factor loadings and mean differences being constrained to be equal across the two age groups, demonstrated adequate goodness-of-fit index to the data. Comparing the baseline and metric models, they showed no changes in the CFI but only showed a RMSEA of less than 0.002, thus supporting the factor loadings invariance across the two age groups. Nevertheless, the comparison between the metric and scalar models indicated over 0.04 differences in CFI and over 0.5 differences in RMSEA, which indicated that there was a mean difference variance across age groups. In conclusion, the results revealed that the factor loadings of the EXSEM with self-compassion were invariant across early and older adolescents. However, the mean differences of EXSEM with self-compassion were somewhat less invariant across age groups compared to that of constrained factor loadings. However, according to Jan-Benedict and Baumgartner (1988), the invariance in the configural and metric models is adequate to determine the validity of the measurement to be applied across groups. Therefore, the EXSEM revised with self-compassion was applicable to early and older adolescents.

### 3.8. Multiple Indicators, Multiple Causes (MIMIC) Structural Equation Modeling (SEM)

Apart from using the path analysis to demonstrate the conceptual model—EXSEM with self-compassion and mental well-being—structural SEM, which includes the latent variables and accounts for the measurement error in the measurement models, was conducted to further investigate the relationship within the EXSEM with self-compassion and mental well-being.

First, according to the suggested scoring of Neff (2003) and Altman (2017), the sum of the subscales of self-compassion and body compassion scales were computed as factor indicators of the scales. The CFA of the respective scales showed adequate goodness of fit, with self-compassion and body compassion at X^2^(8.76/2) = 4.3, CFI = 0.995, TLI = 0.997, SRMR = 0.0128, RMSEA = 0.0564(0.022−0.096), and X^2^ = 0, CFI = 0.913, TLI = 0.914, SRMR = 0.053, RMSEA = 0.026 (0.013 − 0.013). However, the SEM results of the hypothesized conceptual model only showed a marginal fit, with X² (3375.33)/746 = 4.5, CFI = 0.911, TLI = 0.886, SRMR = 0.12, and RMSEA = 0.058 (90% CI = −0.055−0.061). Based on the standardized residual recommendation, the correlation between body compassion, common humanity subscales, self-kindness, and self-compassion–community humanity and between defusion, self-judgment and isolation were identified. However, it could only generalize an acceptable model fit, with X^2^ (3074.3)/741 = 4.14, CFI = 0.921, TLI = 0.899, SRMR = 0.107, and RMSEA = 0.054 (90% CI = 0.053−0.057).

As a result, further exploration of hidden relationships within the model is essential. Hence, MIMIC and SEM were adopted. MIMIC modeling is a special type of SEM that includes both measurement and structural models. In particular, the structural model not only enables the paths to show the causal relationships and effects between latent variables but also integrates with additional variables. MIMIC modeling assumes the influences of the latent factors and their indicators; hence, it includes paths between other covariates and latent factor indicators [62]. The development of these paths was provided by modification indices. The higher the modification indices, the larger the improvement. In the current study, other than the paths originating from the hypothesized EXSEM revised with self-compassion and mental well-being, the MIMIC modeling was applied and discovered a few paths in revealing the effect of well-being on the factor structure of self-compassion. However, it showed a satisfactory goodness-of-fit model, with X^2^(2588.35)/740 = 3.49, CFI = 0.938, TLI = 0.915, SRMR = 0.06, and RMSEA = 0.048 (90% CI = 0.047–0.05). The model, apart from the original hypothesized patterns, it revealed five additional paths between mental well-being and the factor structure of self-compassion and body compassion. It showed that mental well-being positively affects self-kindness, mindfulness, common humanity, and acceptance, while self-compassion positively affects defusion.

## 4. Discussion

This study aimed to examine and investigate the relationship between physical activity and self-compassion based on the proposed EXSEM-SC. The study results indicated that the relationship between physical activity and self-compassion could be demonstrated by the EXSEM-SC, with a satisfactory goodness-of-fit index in the SEMs. The SEM demonstrated indirect paths from physical activity to self-compassion and mental well-being through exercise self-efficacy and body compassion. In addition, the SEM demonstrated direct paths from physical activity to self-compassion, the positive facets of self-compassion and mental well-being, indicating the significant effect of physical activity on self-compassion.

### 4.1. Relationship between Physical Activity and Self-Compassion

The study outcomes have successfully revealed the relationship between physical activity and self-compassion, with both direct and indirect effects. The causal effect between physical activity, physical self-perception, and self-esteem was empirically supported by the EXSEM; hence, the indirect effects of physical activity, body compassion, and self-compassion could be fully explained by the EXSEM with the replacement of body compassion and self-compassion. On the other hand, the model successfully indicated a direct path from physical activity to self-compassion, with β = 0.078, in which it showed a close effect size with the outcomes of the meta-analysis on the relationship between physical activity and self-compassion (r = 0.18) [40]. Other than the results from the meta-analysis, studies that revealed the relationship between physical activity and self-compassion were those that indicated the effect of mindful-related physical activities, such as yoga, on self-compassion and mental well-being [63,64,65]. However, it is noteworthy that less than 1% of the participants reported any yoga or mindful-related physical activity participation in the self-report physical activity questionnaire. Therefore, the study outcomes could further justify the effect of general physical activities, such as running, cycling, basketball, and other leisure physical activities, on self-compassion and mental well-being.

### 4.2. Relationship between Physical Activity and Mental Well-Being Throught Self-Compassion

Moreover, a direct effect between physical activity and mental well-being is expected. Significant studies have revealed the dose-and-response effect of physical activity on reducing mental vulnerability, such as negative self-worth, emotional problems, depression, and anxiety [66,67,68,69] and enhancing positive psychological well-being, such as resilience, global self-worth, physical self-worth, and self-esteem [69,70]. Nonetheless, the study outcomes demonstrated an indirect effect between physical activity and mental well-being through the mediation of self-compassion, which achieved a larger effect size. Studies have also revealed the significant mediating role of mindfulness and self-compassion in body-and-mind activity, quality of life, perceived stress, and well-being [64,71]. Therefore, the direct effect of physical on mindfulness towards self-compassion, as well as the mediating effect of self-compassion on physical activity and mental well-being is supported. Furthermore, providing a stronger effect of physical activity on mental health mediated by self-compassion, physical activity interventions could be promoted to enhance self-compassion and mental health in both non-clinical and clinical groups, thus enriching the significant impact of physical activity as a self-care tool in achieving a positive overall mental well-being.

### 4.3. Influencing Effect of Age on Physical Activity and Self-Compassion

The study results indicated that there were significant differences (in both *t*-test and mean differences model invariance) between early and older adolescents in physical activity level and self-compassion. Furthermore, they showed that age was considered as a significant moderator between physical activity and self-compassion, in which notwithstanding a high physical activity level, individuals’ self-compassion could be reduced because of the increase in age. The existing literature has indicated that physical activity levels and physical activity intentions are less likely to be moderated by age, unless among older adults [72,73,74]. However, despite the study showing no significant correlation between age and self-compassion, there were no significant differences between early and older adolescents (junior and senior forms) in the level of self-compassion. The current study indicated that age has a significant interaction with physical activity and self-compassion. With age interacting with physical activity, physical activity would result in a negative relationship with self-compassion. Studies indicated that adolescents’ self-compassion differed by age and therefore in terms of emotional well-being [75,76], particularly stating that older adolescents tend to show lower self-compassion levels compared to early adolescents. However, older adolescents with high self-compassion levels would show a stronger protective effect on negative affect compared to early adolescents [44,77]. However, this negative association between age and self-compassion has not been applied to adults [78]. Based on the existing literature and the current outcomes, the effect of physical activity may not be seen as stable and strong enough to affect the moderating effect of age on self-compassion. Therefore, these results may enlighten the age at which physical activity interventions could be implemented to facilitate adolescents in maintaining the level of self-compassion and positive mental well-being during their psychosocial development and puberty.

### 4.4. MIMIC SEM

The MIMIC SEM demonstrated a significant relationship between physical activity, self-compassion, and mental health through exercise self-efficacy and body compassion, similar to the hypothesized model and the path analysis model. In addition, it has indicated a direct relationship between physical activity, self-compassion, and well-being, which corresponded with the path analysis results. Hence, it could be interpreted that the measurement error of the measurement model was less likely to affect the indication of the EXSEM with self-compassion and mental well-being. However, the MIMIC model was shown to be a good fit model with an additional path from mental well-being to the indicators of self-compassion and body compassion and from self-compassion to the indicators of body compassion.

First, the MIMIC model has demonstrated a significant pathway from self-compassion to defusion, which indicated that self-compassion facilitates individuals to face their body-related deficiencies instead of isolating or escaping from them. Research has indicated that self-compassion can significantly influence one’s physical well-being [79], body appreciation, and body image [36,80], while it can weaken the relationship between social comparison and body dissatisfaction [81]. Moreover, it is obvious that the conceptual model of body compassion is derived from that of self-compassion; the common humanity and self-isolation factors have been involved in the defusion items (reverse coding) of body compassion. Hence, it can explain the stronger effect of self-compassion on defusion.

Second, the MIMIC model has demonstrated another pathway from mental well-being to body compassion acceptance. Studies examining the causal effect of mental well-being on positive body image or body-related issues are limited. Studies have documented the association and causal effect between body acceptance, positive body image, and mental well-being [82,83,84], which supports the extra direct pathway between body compassion and mental well-being in both the MIMIC model and path analysis; this can be seen as a justly supporting evidence of this path.

Finally, it demonstrated significant pathways between mental well-being and the three positive self-compassion factors: self-kindness, common humanity, and mindfulness. Studies have revealed the association between self-compassion and well-being, while more have been focused on the causal effect of self-compassion on well-being [79,85,86,87], in which it was also indicated in the MIMIC model. Studies have also indicated that self-kindness and positive mental well-being were significantly correlated; however, only the causal effect of self-kindness on mental well-being was further examined and found to be significant [88]. However, only few studies have demonstrated that people with better mental well-being tend to show a higher level of self-compassion and a lower level of self-judgment [89,90]. Moreover, the role of mindfulness on mental well-being has been well documented [91,92]; however, seemingly, only the significant correlation studies between mindfulness and well-being [93] can explain the potential causal effect of mental well-being on mindfulness.

According to the extra pathways indicated in the MIMIC model, it can be interpreted that physical activity can lead to positive body compassion and self-compassion while further affecting one’s mental well-being. However, individuals with positive mental well-being were able to generate positive self-compassion and body acceptance, which was supported by the interrelationship between self-compassion, body image, and mental well-being stated in their significant associations.

## 5. Contribution and Limitation

A few imperfections in the current study are worth noting.

Firstly, because of the coronavirus disease 2019 (COVID-19) pandemic, adolescents’ physical activity levels might be affected, thereby affecting the level of effects on the level of self-compassion and mental well-being.

Secondly, objective physical activity measures were not utilized in the current study. Although the PAQ-C questionnaire was well-developed and validated by existing studies, including objective physical activity measures, such as accelerometers, it could be considered as a dual-purpose test to ensure the quality of the part of the self-reported physical activity. However, because of the COVID-19 pandemic and hygiene issues, objective physical activity measures were allowed in secondary schools. Hence, interventions with objective physical activity measures or well-recorded exercise frequency, duration, and intensity could be implied for future studies to enhance the objectivity of physical activity measures.

Thirdly, despite the current study having adopted the Self-Compassion Scale-Chinese Version, the measurement model was not shown as fit until item 7 and item 8 were deleted. Item 7 (Common Humanity) indicates, “When I’m down and out, I remind myself that there are lots of other people in the world feeling like I am.” and Item 8 (Self-judgement) indicates, “When times are really difficult, I tend to be tough on myself.” Yet, it was less likely to be explained by existing literature. However, providing the current sample had a moderate level of self-compassion, it is less likely they would impose judgmental thoughts on themselves. Moreover, in a qualitative study extended from the EXSEM-SC model [94], the results indicated that Hong Kong secondary school students would go through a mindful self-reflection process before imposing any critical judgement on themselves, nevertheless, most of the participants in the qualitative study showed kindness towards their self-reflection. Moreover, the study has also displayed the fact that Hong Kong secondary school students tended to seek help from their peers or relate their own situation with the peers around them, instead of people whom they do not know or people from the outside world in general. Hence, it partially supported the possible reasons for the need to delete the respective items in the current sample.

Fourth, it is essential to clarify that the EXSEM-SC has provided an alternative angle for the relationship between physical activity and self-compassion. However, concerning the original Exercise and Self-esteem Model that has been strongly developed and widely utilised in the field, it is vital to conduct a further examination comparing the significance and effectiveness of the two constructs. This would enhance the solidarity of the model revised with self-compassion as well as to further distinguish between the effect of physical activity on self-compassion and self-esteem.

Finally, providing the significant pathways between physical activity and the positive facets of self-compassion indicated the dose-and-response effect of physical activity on positive self-compassion, in particular; it did not justify the effect on reducing negative self-compassion. Therefore, further investigation, with a qualitative approach, in particular, is needed to explore the latent relationships between physical activity and each facet of self-compassion.

Nevertheless, the current study has shown a significant contribution to the field of physical activity and mental health studies in Hong Kong. First, the current study translated the body compassion scale into Chinese and validated among Hong Kong adolescents, which has significantly revealed the relationship between body-part issues and mental well-being in a non-Western country. The results of the body compassion scale revealed that Hong Kong adolescents were less likely to seek help from others when facing body-related issues and tend to face the problem by themselves instead. Despite being a hit research topic in Hong Kong, the most relevant study was published a decade ago. Therefore, the investigation of body compassion has successfully indicated how Hong Kong adolescents perceive and deal with their body-related issues. Second, despite recent studies reporting the prevalence of insufficient healthy lifestyle behavior among Hong Kong children and adolescents [95], there is a lack of updated cross-sectional studies that investigate the relationship between physical activity and mental health among Hong Kong adolescents and secondary school students. Therefore, the current study was able to provide an overview of Hong Kong adolescents’ self-reported physical activity, mental well-being, and self-compassion status. Furthermore, owing to the descriptive results, it showed an insufficient physical activity level and an average level of self-compassion among Hong Kong adolescents; hence, it supports the importance of promoting engagement in physical activity, at the same time, embracing physical activity as a self-care tool to enhance adolescents’ self-compassion and overall mental well-being. Finally, the significant direct effect between physical activity and self-compassion and the adequate goodness-of-fit model support the hypothesized conceptual framework of EXSEM revised with self-compassion, in which self-compassion can be improved through engagement in physical activity. At the same time, it echoes the does-and-response effect of physical activity in achieving positive self-compassion and mental well-being [11,12,13], thus releasing psychological tension among Hong Kong adolescents. Hence, the elements of self-compassion should contribute to future physical activity interventions. In addition, because of the COVID-19 pandemic, both healthy lifestyle behaviors and mental well-being of Hong Kong students were highly affected; hence, home-based physical activity intervention programs should be promoted to enhance Hong Kong adolescents’ ability to show compassion toward oneself and the ability to exercise stay-at-home self-care.

## 6. Conclusions

By revealing the relationship between physical activity and self-compassion, the current research has manifested the development of the conceptual framework modified from the Exercise and Self-esteem Model. Based on the results of the Exercise and Self-Esteem model revised with self-compassion, it was able to justify that physical activity participation can act as a tool to improve self-compassion, as well as mental well-being. The development and investigation of the EXSEM-SC have provided a manifestation of a construct that physical activity could improve self-compassion and aid students’ mental health. This justification could also support a theoretical-based design physical activity intervention to improve secondary school students’ self-compassion, thus enhancing mental health or easing mental illness.

## Figures and Tables

**Table 1:** Descriptive statistics.

		Minimum	Maximum	Mean	Std. Deviation
Age		11.00	16.00	14.13	1.45
		Frequency	Percent		
Gender					
	Women	376	35.0		
	Men	698	65.0		
Family Monthly Income	2000 or below	83	7.7		
	20,000–30,000	108	10.1		
	30,000–40,000	103	9.6		
	40,000–50,000	73	6.8		
	50,000 or above	214	19.9		

**Table 2:** Mean of the model variables.

Variables	Mean	Std. Deviation
Self-Compassion	3.0886	0.49
Body Compassion	3.1144	0.50
Exercise Self-efficacy	4.7856	2.32
Mental Well-being	3.3198	0.87
Physical Activity	2.3023	0.94

**Table 3:** Correlation matrix of the model variables.

Variables	SC	BC	ESE	WB	PA
BC	0.546 **	−			
ESE	0.189 **	0.186 **	−		
WB	0.481 **	0.391 **	0.260 **	−	
PA	0.119 **	0.089 **	0.505 **	0.229 **	−
Age	−0.033	−0.105 **	−0.087 **	−0.057	−0.147 **
Income	0.079	0.072	0.075	0.079	0.107 *

Note: SC = self-compassion; BC = body compassion; ESE = exercise self-efficacy; WB = mental well-being; PA = physical activity; * *p* < 0.05; ** *p* < 0.01.

**Table 4:** Summary of goodness-of-fit measurement models.

Measurement	Chi-Squared Test		Indices			
	X^2^/df	*p*	CFI	TLI	SRMR	RMSEA
Self-compassion	4.2	<0.001	0.920	0.907	0.044	0.055
Body Compassion	3.36	<0.001	0.944	0.938	0.042	0.056
Exercise Self-efficacy	5.7	<0.001	0.954	0.945	0.031	0.066
Mental Well-being	4.6	<0.001	0.920	0.981	0.017	0.054
Physical Activity	2.15	<0.001	0.995	0.992	0.020	0.033

Note: CFI = comparative fit index; TLI = Tucker–Lewis index; SRMR = standardized root mean square residual; RMSEA = root mean square error of approximation, *p* = *p-*value; X^2^ = chi-square, df = degree of freedom.

**Table 5:** Standardized direct, indirect, and total effect of physical activity on self-compassion.

Pathways	Direct Effect	Indirect Effect	Total Effect
Physical Activity→Exercise Self-Efficacy→Body Compassion→Self-Compassion	−	0.049 ***	−
Physical Activity→Self-Compassion	0.078 **	−	0.127 **
Body Compassion→Self-Compassion	0.505 ***	−	−

** *p* < 0.01, *** *p* < 0.001.

**Table 6:** Standardized direct, indirect, and total effect of physical activity on mental health.

Pathways	Direct Effect	Indirect Effect	Total Effect
Physical Activity→Exercise Self-efficacy→Body Compassion→Self-Compassion→Mental Well-Being	−	0.018 ***	−
Physical Activity→Mental Well-Being	0.167 ***	−	0.659 ***
Body Compassion→Mental Well-Being	0.175 ***	−	0.571 ***
Self-Compassion→Mental Well-Being	0.396 ***	−	−

*** *p* < 0.001.

**Table 7:** Summary of goodness of fit of the measurement invariance analysis models.

EXSEM with SC	Chi-Squared Test		Indices			
	X^2^/df	*p*	CFI	TLI	SRMR	RMSEA(90%CI)
Baseline Model	3.3	<0.001	0.987	0.956	0.027	0.066 (0.038–0.10)
Metric Model	3.3	<0.001	0.987	0.956	0.027	0.068 (0.038–0.10)
Scalar Model	6.6	<0.001	0.995	0.900	0.065	0.103 (0.08–0.127)

Note: *N* of early adolescents = 591, *N* of older adolescents = 483.Abbreviations: EXSEM with SC = exercise and self-esteem model revised with self-compassion; CFI = comparative fit index; TLI = Tucker–Lewis index; SRMR = standardized root mean square residual; RMSEA = root mean square error of approximation, *p* = *p*-value; X^2^ = chi-square, df = degree of freedom.
